# Supplementary material for: Organoid-based in vitro system and reporter for the study of Cryptosporidium parvum sexual reproduction
Source: Microbiol Spectr. 2025 Jun 25;13(8):e00502-25. doi: 10.1128/spectrum.00502-25 (PMC12323322; doi:10.1128/spectrum.00502-25)
Supplement: Supplemental figures — Figures S1 to S4. [file spectrum.00502-25-s0001.pdf]

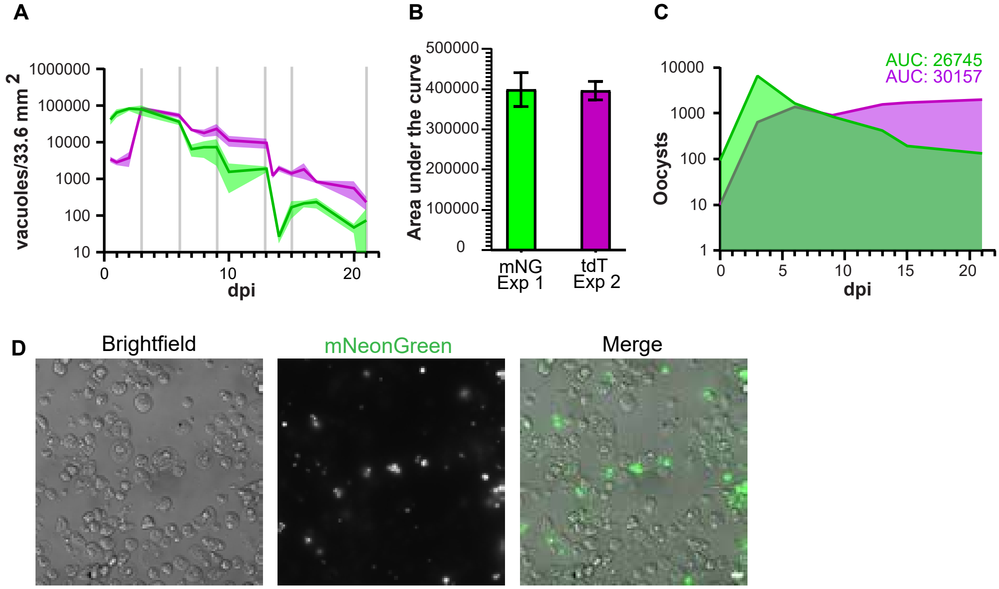

Supplementary Figure 1 ODM infection supports long term infection and oocyst production. (A) Vacuole counts from ImageJ macro analysis of fluorescent parasite vacuoles adjusted for total cell culture area. Each colored line represents a different experiment. mNG infection (green) and tdT infection (magenta). Vertical grey lines show days with supernatant collection; images were collected before supernatants were collected. Standard deviation is shown as shaded area surrounding each line. **(B)** Area under the curve (AUC) of experiments in A. **(C)** Flow cytometry quantification of oocysts collected from ODM supernatant based on FSC and SSC of purified WT stock oocysts and expression of mNG or tdT. **(D)** Images of supernatant collected from ODMs infected with mNeonGreen expressing parasites 6 dpi. Dead host cells that have sloughed off the monolayer can be observed as well as various parasite stages (green). Parasites are observed to still be attached to host cells in many cases. Scale bar is 5 µm.

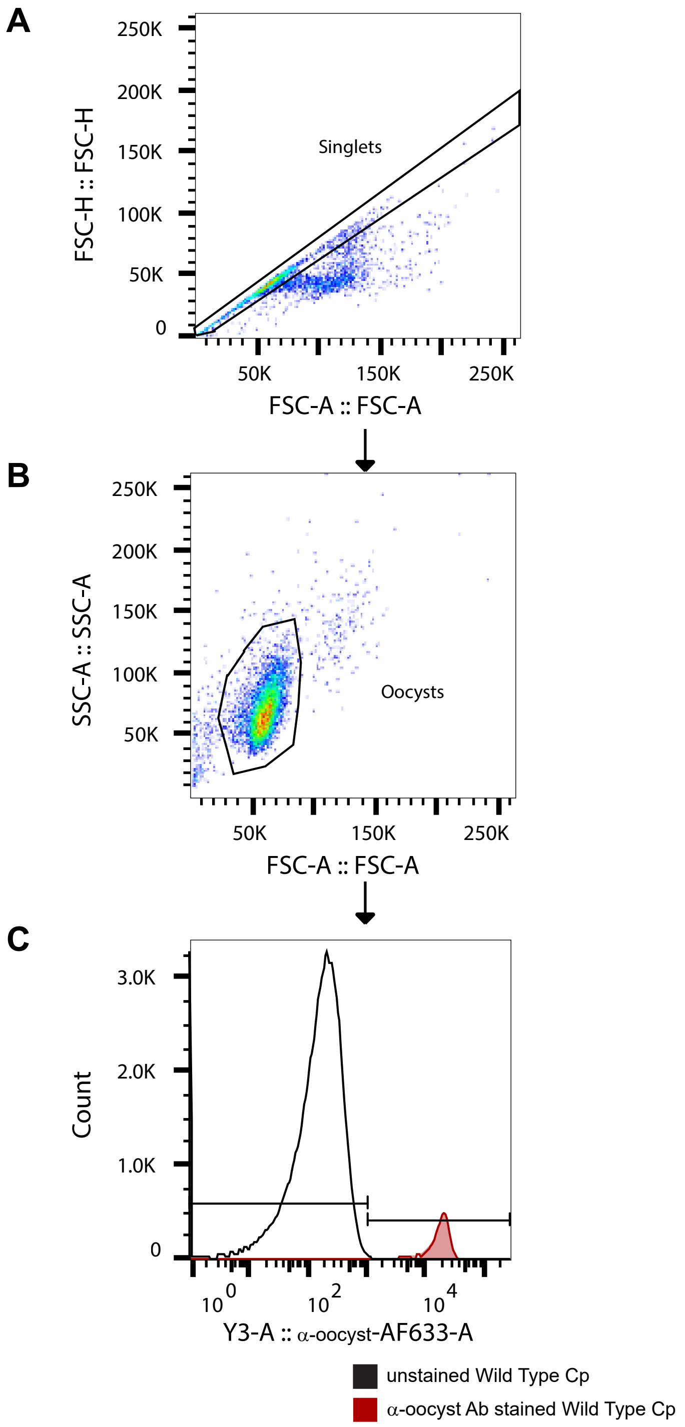

Supplemental Figure 2. Gating strategy for oocysts used for data in figure 3. (A) Doublet discrimination was performed, then **(B)** FSC and SSC were used to find oocyst size and granularity based on WT stock oocysts. **(C)** Fluorescence histograms validating the ability to identify oocysts by α-oocyst wall monoclonal Ab staining. WT oocysts were stained with α-oocyst wall monoclonal Ab or left unstained and then mixed together to generate the histograms from a single sample. This figure was prepared with FlowJo and Adobe Illustrator.

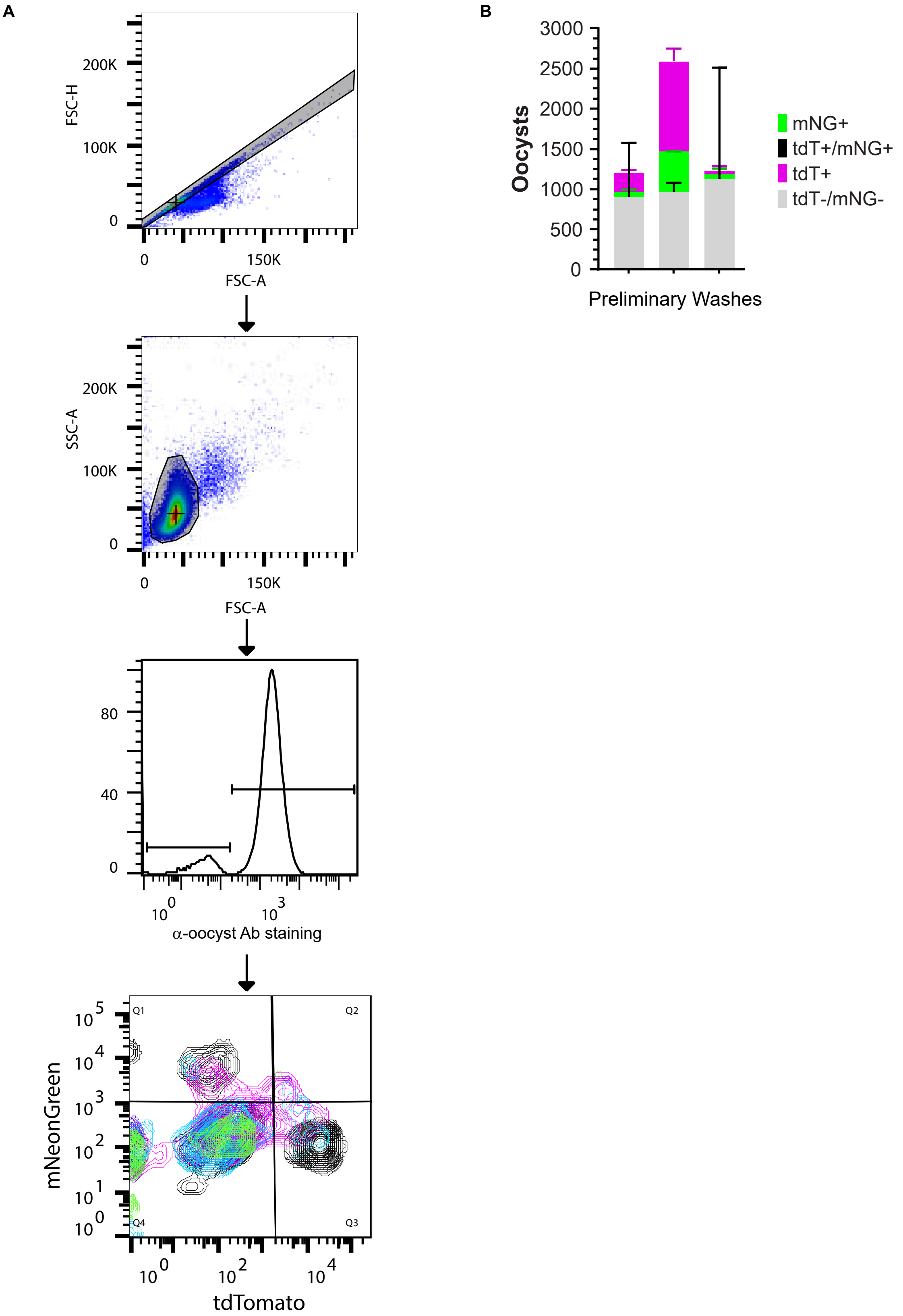

Supplemental Figure 3. Gating strategy for figure 4. (**A**) Doublet discrimination followed by gating on FSC and SSC based on purified stock WT oocysts, then α-oocyst wall monoclonal antibody staining to identify oocysts. To assess the subpopulations of oocysts mNeonGreen and tdTomato expression was used to identify single positive, double positive, and double negative populations. Contour plots of samples with mixed populations are shown in different colors in the last panel. (**B**) Oocyst counts from the washes done 3 hpi to remove unexcysted oocysts and unattached parasites. Washes show that either the tdT or mNG oocyst stock was either contaminated with WT or reverted to WT at some point. The mixed population of nonfluorescent and mNG oocysts in the mNG oocyst stock was visually confirmed by looking at the stock microscopically. Visual inspection of the tdTomato stock showed a pure population. This explains why the majority of the PVs counted by ImageJ macro in Figure 4 were tdT positive and that the macro counts significantly underestimate the PV count in the ODM. There was also likely crossing between WT and mNG or tdT that could not be distinguished from tdT x tdT or mNG x mNG crossing in this assay.

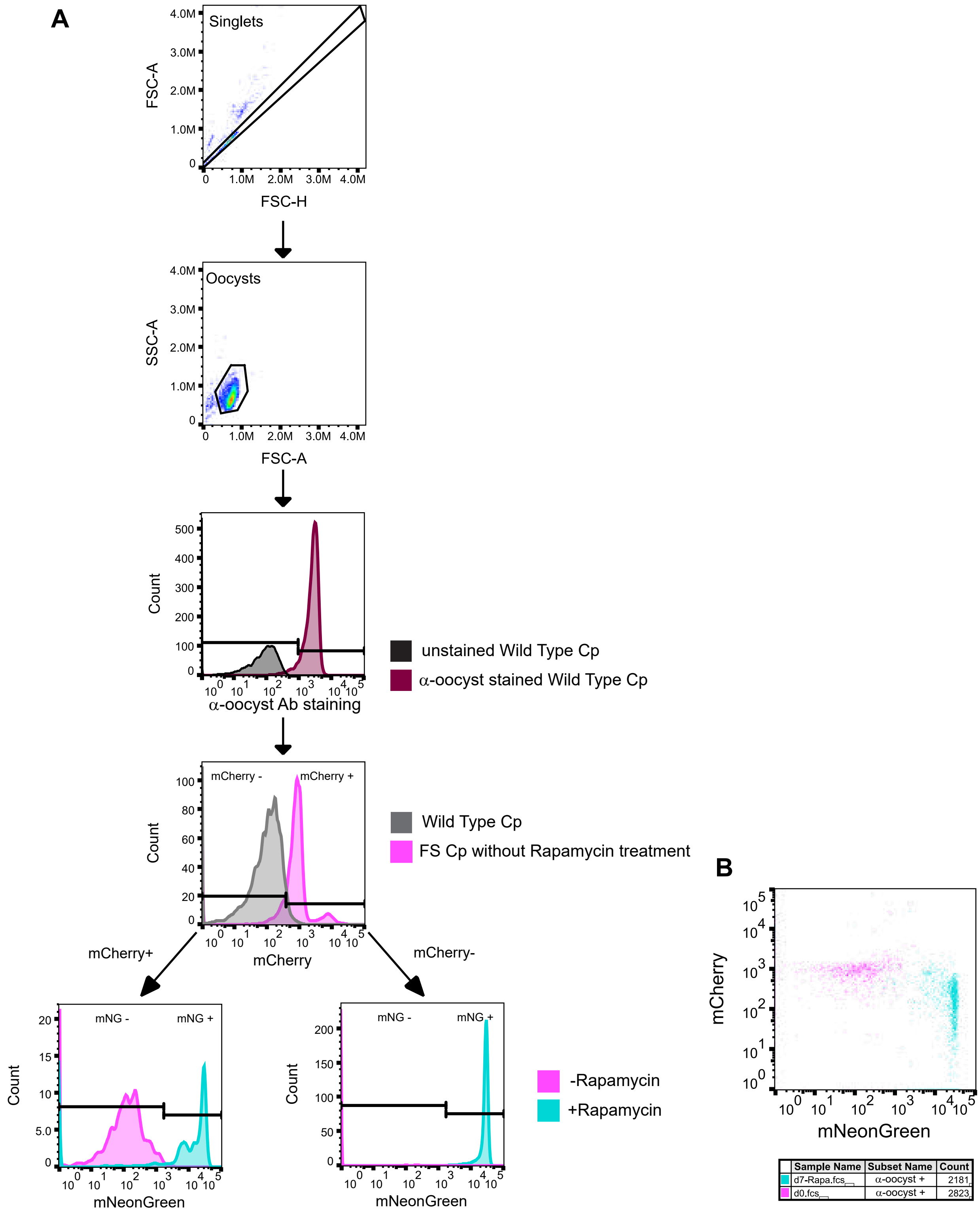

Supplemental Figure 4. FS Oocyst gating strategy. (**A**) Doublet discrimination followed by FSC/SSC gating based on purified stock oocysts. Then then α-oocyst wall monoclonal antibody stained populations were gated based on expression of mCherry or mNeonGreen. The final two histogram plots show oocysts from the untreated mouse (pink) and the treated mice (teal). mNeonGreen gates were set based on nonfluorescent stock oocysts being negative. (**B**) Oocysts from untreated mice are shown with pink dots and oocysts from mice that received rapamycin are shown in teal dots. mCherry expression is on the y-axis and mNeonGreen is on the x-axis.
