# Supplementary material for: Organoid-based in vitro system and reporter for the study of Cryptosporidium parvum sexual reproduction
Source: Microbiol Spectr. 2025 Jun 25;13(8):e00502-25. doi: 10.1128/spectrum.00502-25 (PMC12323322; doi:10.1128/spectrum.00502-25)
Supplement: Supplemental text — Materials list. [file spectrum.00502-25-s0002.docx]

# Materials List

| **Material** | **Vendor** | **Product #** |
| --- | --- | --- |
| Advanced DMEM/F12 | Gibco | 12634028 |
| Nicotinamide | Millipore | 481907 |
| N-acetylcysteine | Sigma | A9165 |
| mouse epidermal growth factor | R&D Systems | 2028-EG |
| Penicillin/streptomycin | Corning | 30-002-Cl |
| GlutaMax | Gibco | 35050061 |
| HEPES | Gibco | 15630-080 |
| N2 supplement | Gibco | 17502048 |
| B27 supplement | Gibco | 17504044 |
| Matrigel™ | Corning | CB40234 |
| Y27632 inhibitor | R&D Systems | 125450 |
| SB 431542 | R&D Systems | 16141 |
| A83-01 | Sigma | SML0788 |
| SB 202190 | SYNthesis | SYN-1073 |
| 0.25% Trypsin/EDTA | Gibco | 25200-056 |
| High-Capacity RNA-to-cDNA Kit | Applied Biosystems | 4387406 |
| RNeasy | QIAGEN | 74104 |
| PowerUp Mastermix | Applied Biosystems | 4367659 |
| QIAmp DNA mini Kit | Qiagen | 51306 |
| ThinCert Tissue Culture inserts | Greiner Bio-one | 662 641 |
| Fetal bovine serum, H.I. | Sigma | 12306C, Lot 17H093 |
| Fluorogel | Electron Microscopy Science | 17985-10 |
| Prolong Antifade Gold | Invitrogen | 936941 |
| EDTA (pH 8.0) | Promega | V4231 |
| 4% Paraformaldehyde | ThermoScientific | J19943-K2 |
| DAPI | AppliChem | A4099, 0010 |
| Hoescht | AnaSpec Inc. | AS-83219 |
| Bovine Serum Albumin | Sigma | A7906 |
| Triton-X-100 | Promega | H5142 |
| Tween 20 | Fisher Bioreagents | BP337-500 |
| MicroAmp Optical Reaction plates | Applied Biosystems | 4309849 |
| 1.2 µm Syringe filter | Sartorius | 17593 |
| 40 µm nylon Cell strainer | BD Falcon | 352340 |
| GeneArt™ Gibson assembly HiFi Cloning Kit | ThermoFisher Scientific | A46626 |

| **Cell lines** | | |
| --- | --- | --- |
| L-WRN | ATCC (Miyoshi et. al 2013) | CRL-3276 |
| C57Bl6/NJ intestinal stem cells | Isolated in Huston lab | NA |
| HCT8 |  |  |

| **Equipment** |  |  |
| --- | --- | --- |
| MACSQuant VYB | Miltenyi Biotec | Harry Hood Bassett Flow Cytometry and Cell Sorting Facility, UVM |
| CytekAurora | Cytek | Harry Hood Bassett Flow Cytometry and Cell Sorting Facility, UVM |
| QuantStudio6 | Applied Biosystems | Vermont Integrative Genomics Resource DNA Facility, UVM |
| JEOL 1400 transmission electron microscope | JOEL | Microscopy Imaging Center, UVM |
| Lonza 4D Nucleofector Nucleofection System | Lonza | NA |
| Ti Eclipse | Nikon | NA |

| **Parasites** | |
| --- | --- |
| *C. parvum* | Bunchgrass Farms |
| tdTomato *C. parvum* | gift from Striepen Lab |
| mNeonGreen *C. parvum* | gift from Striepen Lab |
|  |  |
| **Software** | |
| ImageJ 1.53k | NIH |
| Adobe Illustrator | Adobe |
| FlowJo 10.8.0 | BD |
| QuantStudio 1.3 | Applied Biosystems |
| MS Excel 2010 | Microsoft |
| GraphPad Prism 10 | GraphPad |

| **Antibodies/stains** | **Host** | **Source** | **Product #** |
| --- | --- | --- | --- |
| Anti-ChgA | mouse | Proteintech | 60135-1-Ig |
| Anti-Lysozyme | rabbit | Proteintech | 15013-1-AP |
| Anti-Muc2 | rabbit | Proteintech | 27675-1-AP |
| Anti-ZO-1 | mouse | Invitrogen | 33-9100 |
| *C. parvum* oocysts monoclonal antibody | mouse | Invitrogen | MA183095 |
| anti-Mouse, Alexa Fluor 488 | Goat | LifeTechnologies | A11029 |
| anti-Rabbit, Alexa Fluor 488 | Donkey | LifeTechnologies | A21206 |
| Alexa Fluor 633 phalloidin | NA | Invitrogen | A22284 |
| FITC-VVL | NA | Vector Labs | FL-1231 |
| Anti-mNeonGreen | Mouse | Chromotek/Proteintech | 32F6 |
| Anti-RFP | Rabbit | Rockland Immunochemicals | 600-401-379 |

|  | **Target** | **Sequence 5'-3'** | **PrimerBank ID/Reference** |
| --- | --- | --- | --- |
| housekeeping | mGapdh FWD | AGGTCGGTGTGAACGGATTTG | 6679937a1 |
|  | mGapdh REV | TGTAGACCATGTAGTTGAGGTCA |  |
| stem cell | mLgr5 FWD | CCTACTCGAAGACTTACCCAGT | 6753842a1 |
|  | mLgr5 REV | GCATTGGGGTGAATGATAGCA |  |
| enteroendocrine | mChgA FWD | ATCCTCTCTATCCTGCGACAC | 6680932a1 |
|  | mChgA REV | GGGCTCTGGTTCTCAAACACT |  |
| goblet cell | mMuc2 FWD | GCTGACGAGTGGTTGGTGAATG | Holthaus et al., 2021 |
|  | mMuc2 REV | GATGAGGTGGCAGACAGGAGAC |  |
| tight junction | mZo1 FWD | GCCGCTAAGAGCACAGCAA | 6678355a1 |
|  | mZo1 REV | TCCCCACTCTGAAAATGAGGA |  |
| *C. parvum* | 18S rRNA FWD | TAGAGATTGGAGGTTGTTCCT | NA |
|  | 18S rRNA REV | CTCCACCAACTAAGAACGGCC |  |

**Primers**

| **Purpose** | **Sequence 5'-3'** | **PrimerBank ID/Reference** |
| --- | --- | --- |
| For mCherry excision assessment | Cre60 FWD | CTATTTATTCTGCCGGGTCAG |
|  | cgd1_3020 REV | GAGATTTTTCACCAATTTCTACTTTATC |
| For G block 8 creation | Cre60 FWD | CTATTTATTCTGCCGGGTCAG |
|  | nluc REV | ATCACCTTAAAGTGATGATC |

**FS G-blocks**

|  | **G-blocks for Gibson Assembly** | | |
| --- | --- | --- | --- |
|  | **Name** | **Length (bp)** | **Sequence** |
| **Fertilization Switch** | **GB_12** | 1075 | CAGGAAACAGCTATGACCATGATTACGCCAAGCTTGCATGCCTGCAGGTCTTTAAAGCAATAATATCACTCATACCTACTGCAAATAAGATTGGAAATACGCTAGGAATTAAGATAAAAAGAAAAACTTAATCGATACTATCCTACACGCCACGAACTTATTATTAATTCAATTTACACCACGCAGCCCAGGATCTGCATACAGATAATAACATTTTCCATGTATGTTCAGAAAATGCTTGCCTGTCCCTAAACATGTCCATCAGATTTTTCCTGACTTCATCTGAGGTGGATCCATTGCTAGCACCCGGGTTGCTAGGTGAAGCTTCTAATTTTAGAAGCTCTACATCAAAGACGAGAGTTGCATGTGGTGGAATTATACCTGGGTGTCCAGTGGCACCATAAGCATAATCTGGAGATATAGTCAATTTTGCTCTTTGTCCTACACTCATTTGAGCAACTCCTTCTTCCCAGCCTCGAATAACCTCTTGCTTGCCTAGCATAAATTTAAAGGGCTTATTTCTGTCCCGACTTGAATCGAATTTTTTTCCATCTTCAAGCATCCCGGTGTAATGCACAACACATGTCTGGCCACGTTTAGGAAACGTGCGCCCATCTCCTGGCGAGATTGTTTCAACTTGTACTCCTCTAGAAACTTTTCTTTTCTTTTTAGGTGCCATGGTGGCAATAAAAGAAAAAACTATTTAATGAAATTGACCTAATAATTAAATTAGGGCAGATATTCAAATAGTAATTGAAATTATTAGGTAAAAAATTGTTGATTGAGGAAAAACAATAATTCGAGGATACACCTAACGCATATACGATACAATTTACACAAATTCGTGAGTGCTCATATTAGAGTTTAATCTAAATTGTTTTAAAGAAACATATGATAATAAACTATCGCTTTGCAATAAATTAAAATATTGAACTTTCGTTGGAATGTGTTTTTTCAATAATTTTTGATCGGATTGTAAATTTATTGGATAAAACCAATCAATTTTTTGGGCGCAATTTTCAGGAAAATCAAACTATTAATTTAGAGATTGATTAAATAATTTTTAATTGACATA |
|  | **GB_13** | 1186 | ATTAAGAGGCACTAATTTTCCTTCCGATGGGCCCGTAATGCAAAAAAAGACAATGGGATGGGAAGCATCATCCGAGCGGATGTATCCAGAGGATGGGGCACTGAAAGGCGAAATTAAACAAAGGTTAAAACTTAAGGATGGAGGACATTACGATGCTGAAGTCAAAACCACATACAAAGCAAAAAAGCCTGTTCAGCTACCAGGTGCATATAACGTCAATATAAAATTGGATATCACCAGCCACAATGAAGATTATACAATAGTAGAACAATATGAAAGAGCAGAGGGTAGACATTCAACTGGTGGGATGGATGAATTGTATAAATAAATAACTTCGTATAGCATACATTATACGAAGTTATGCTAGCATGGTTAGTAAAGGAGAAGAAGACAATATGGCAAGCTTGCCAGCTACCCATGAACTTCACATTTTTGGATCAATTAATGGTGTTGATTTTGATATGGTTGGTCAAGGAACTGGAAACCCAAACGACGGTTACGAAGAACTTAATTTAAAATCAACTAAAGGTGATTTGCAATTCTCACCTTGGATATTAGTACCACATATTGGGTACGGATTCCATCAATATTTACCTTATCCAGATGGGATGTCTCCATTCCAAGCAGCAATGGTCGACGGATCAGGATATCAGGTACATAGAACCATGCAATTTGAGGACGGTGCATCACTAACAGTTAATTATAGATATACTTATGAAGGCAGTCACATTAAGGGCGAAGCACAAGTTAAAGGGACAGGTTTCCCAGCCGATGGTCCAGTTATGACTAATTCTTTAACTGCCGCTGATTGGTGTCGATCTAAAAAAACATATCCTAATGATAAGACTATTATTTCAACTTTTAAATGGTCATATACTACAGGCAACGGAAAAAGATATAGGAGTACAGCTAGGACTACTTATACTTTTGCAAAACCAATGGCTGCCAATTACTTAAAAAATCAACCGATGTATGTTTTCCGTAAAACGGAATTGAAACACTCAAAAACTGAATTGAATTTCAAAGAATGGCAGAAAGCATTTACTGACGTTATGGGAATGGATGAATTATATAAATAATAATAAGTTCGTGGCGTGTAGGATAGTATCGATTAAGTTTTTCTTTTTATCTTAATTTGGGGAAACTAAATATACTGAAATTCGGTAGATTCTATATCTCACGGGAC |
|  | **GB_3** | 598 | TAATAAGTTCGTGGCGTGTAGGATAGTATCGATTAAGTTTTTCTTTTTATCTTAATTTGGGGAAACTAAATATACTGAAATTCGGTAGATTCTATATCTCACGGGACAGCTTTTCACACACACTTAGTTCTATATTGCGTCATAACTTTTGTTTTTTTTTGTTGCACTTTTTTTCTCTTATATTCAAGTAAGTGGTTTAGATTCTCTAAGGGCGGGAATATGAATTAGTGGCAATTCAAAGGATTTAAACGATCAGGCGCCTGCACACCAAACTCTAATTCGTACACAGCATGCCCGAGGTTATAGATATAGATACTGCGAAATTATTTTCATTGTTTCAGTTAAGAAATAATAAAATATTTTATTATAGTTATTTTCCAAATTTATTTGAGATTTTTGTATTGAAGTTTAGCCGTCGACATGGTCTTCACACTCGAAGATTTCGTTGGGGACTGGCGACAGACAGCCGGCTACAACCTGGACCAAGTCCTTGAACAGGGAGGTGTGTCCAGTTTGTTTCAGAATCTCGGGGTGTCCGTAACTCCGATCCAAAGGATTGTCCTGAGCGGTGAAAATGGGCTGAAGATCGACATCCATG |
|  | **GB_5** | 1355 | CCAAAGGATTGTCCTGAGCGGTGAAAATGGGCTGAAGATCGACATCCATGTCATCATCCCGTATGAAGGTCTGAGCGGCGACCAAATGGGCCAGATCGAAAAAATTTTTAAGGTGGTGTACCCTGTGGATGATCATCACTTTAAGGTGATCCTGCACTATGGCACACTGGTAATCGACGGGGTTACGCCGAACATGATCGACTATTTCGGACGGCCGTATGAAGGCATCGCCGTGTTCGACGGCAAAAAGATCACTGTAACAGGGACCCTGTGGAACGGCAACAAAATTATCGACGAGCGCCTGATCAACCCCGACGGCTCCCTGCTGTTCCGAGTAACCATCAACGGAGTGACCGGCTGGCGGCTGTGCGAACGCATTCTGGCGGCTAGCATGATTGAACAAGATGGTTTACACGCTGGTTCTCCCGCCGCTTGGGTCGAAAGACTTTTCGGTTATGACTGGGCTCAACAAACCATCGGTTGCTCTGATGCCGCCGTCTTCCGTCTTTCTGCTCAAGGTCGTCCTGTTCTTTTCGTCAAGACCGACCTTTCTGGTGCCCTTAATGAACTTCAAGATGAAGCTGCCCGTCTTTCTTGGCTTGCCACCACCGGTGTTCCTTGCGCTGCTGTCCTTGACGTTGTCACTGAAGCCGGTAGAGACTGGCTTCTTTTAGGTGAAGTCCCCGGTCAAGATCTTCTTTCTTCTCACCTTGCTCCTGCCGAAAAAGTTTCTATCATGGCTGATGCTATGCGTCGTCTTCATACCCTTGATCCCGCTACCTGCCCTTTCGACCACCAAGCCAAACATCGTATCGAACGTGCTCGTACTCGTATGGAAGCCGGTCTTGTCGATCAAGATGATCTTGACGAAGAACATCAAGGTCTTGCCCCTGCCGAACTTTTCGCCAGACTTAAGGCCCGTATGCCCGACGGTGAAGATCTTGTCGTCACCCATGGTGATGCCTGCTTACCCAATATCATGGTTGAAAATGGTCGTTTTTCTGGTTTCATCGACTGTGGTCGTCTTGGTGTCGCCGACCGTTATCAAGATATTGCCTTAGCTACCCGTGATATTGCTGAAGAACTTGGTGGTGAATGGGCTGACCGTTTCCTTGTCCTTTACGGTATCGCCGCTCCCGATTCTCAACGTATCGCCTTCTATCGTCTTCTTGACGAATTCTTCTGATAATAAGTTCGTGGCGTGTAGGATAGTATCGATTAAGTTTTTCTTTTTATCTTAATTTTAATTAAGGCAAAATTTGGCGCAGCTATGGCGCCTCTTGAAAGTGCCTAAAAAGGAGGACTCTAGAGGATCCCCGGGTACCGAGCTCGAATTCACTGGCCGTCGTTTTACA |

|  | | **G-blocks for Gibson Assembly** | | | | |
| --- | --- | --- | --- | --- | --- | --- |
|  | | **Name** | **Length (bp)** | | **Sequence** | |
| **Fertilization Switch** | **GB_7** | | | 1256 | TGAGAGAAAATTTAAAAATTTAAGATGAAAGAAGAATAAAATAATATATAGCCACCATGGCACCAAAAAAGAAAAGAAAAGTTTCTAGAATACTGTGGCATGAAATGTGGCATGAAGGCTTGGAAGAGGCATCTCGTTTGTATTTTGGGGAAAGGAATGTAAAAGGAATGTTTGAGGTTTTAGAACCGTTGCATGCTATGATGGAACGGGGACCCCAAACTTTAAAAGAAACATCATTTAATCAGGCATATGGTCGAGATTTAATGGAAGCACAAGAGTGGTGTAGGAAATATATGAAATCAGGAAATGTCAAGGATCTACTACAAGCGTGGGATCTATATTATCATGTATTCCGACGAATATCAGCTAGCCCCAGCAACCCTGGCGCTAGCAATGGATCCAATAGGAAATGGTTCCCTGCTGAACCAGAGGATGTAAGGGATTACCTATTGTATTTACAAGCAAGAGGACTTGCTGTTAAAACGATACAACAGCACTTGGGCCAGCTAAACATGTTGCATAGGAGAAGTGGATTACCAAGACCTTCTGATTCAAATGCTGTTTCCCTTGTGATGAGGAGAATAAGAAAAGAAAATGTTGATGCTGGAGAGAGAGCAAAACAAGCTTTGGCATTTGAACGCACTGATTTTGACCAAGTCAGATCATTAATGGAGAATTCTGATAGATGTCAGGATATCAGGAACCTCGCATTCTTGGGAATTGCCTACAATACTTTGTTAAGAATTGCAGAAATTGCAAGAATTAGAGTGAAAGATATATCCCGCACAGATGGAGGAAGAATGTTAATCCATATTGGCAGGACTAAGACACTTGTTTCAACAGCTGGTGTGGAAAAAGCATTATCCCTTGGGGTTACTAAATTAGTTGAAAGATGGATAAGTGTTTCTGGAGTAGCTGATGACCCGAATAACTATTTATTCTGCCGGGTCAGAAAAAATGGTGTAGCTGCACCAAGTGCCACCTCACAATTATCCACCCGGGCATTAGAAGGAATATTTGAGGCAACACACCGCCTTATTTATGGAGCAAAAGATGACTCTGGACAAAGATATTTAGCATGGTCTGGACATAGTGCAAGAGTAGGTGCTGCCAGGGACATGGCCAGGGCTGGTGTGTCGATCCCAGAAATTATGCAAGCTGGTGGCTGGACTAATGTTAATATTGTAATGAACTACATTAGAAATTTGGACTCTGAGACTGGGGCCATGGTTAGGTTGCTAGAGGATGGGGACTAA |  |
|  | ***GB_8*** | | | 1043 | CTATTTATTCTGCCGGGTCAGAAAAAATGGTGTAGCTGCACCAAGTGCCACCTCACAATTATCCACCCGGGCATTAGAAGGAATATTTGAGGCAACACACCGCCTTATTTATGGAGCAAAAGATGACTCTGGACAAAGATATTTAGCATGGTCTGGACATAGTGCAAGAGTAGGTGCTGCCAGGGACATGGCCAGGGCTGGTGTGTCGATCCCAGAAATTATGCAAGCTGGTGGCTGGACTAATGTTAATATTGTAATGAACTACATTAGAAATTTGGACTCTGAGACTGGGGCCATGGTTAGGTTGCTAGAGGATGGGGACTAATAATAAGTTCGTGGCGTGTAGGATAGTATCGATTAAGTTTTTCTTTTTATCTTAATTAATACTAATTCTTTTGAATTTCCACATATCTAATCATAATACTAGTCTCTAACATATAAGACTCATTTCTCTGGAGAAAGGTACATATATATTGGAGCAATTCATTATGGGATTCACACTCCCTCTACCCAATATTATCAATAATACAGATAATCAACAATTAGTAGTAGCATTTCTAAATGGTCTACTAGCAGGAATATGTCATTAAATTTTCTAACAATCTATTAAATTATTGTGATTCTCTAAGATATTTGTGTTCCACTTATTTTATTAAAAACTAATATTTTTACTTGGAAGTATTTTCTTTTGCTATTTCTTAACTCAGATATACCATTCCCTACGACTTGTGAAATATTTTACTAATACTTAACTAAAACTATACAAGATTTTTTCCCACTCAAGTTCTAATCAATGCTTACTTTAAACACGTTTTTTTACACCTATATATACTTTAATATTTGTTTTCAATATTTTTATTTATTTGCATGCAAATTATATATCTAAAAATGGCGCCACAATGGCTGGTATATAATATCCAGCCTGCGAGCATGCTTCAGATTTAAAGGAAAAAGGGAGAAACAGCACAAAATTTGTACTTGTTAGAATCGCAAATAAATTTTGTTAAAAAAATTTGATAAAGTAGAAATTGGTGAAAAATCTC |  |
|  | **GB_9** | | | 517 | AAAGTAGAAATTGGTGAAAAATCTCATTTTATTTGTTTCAAAAGAAAAAAATAACTTCGTATAGCATACATTATACGAAGTTATATGGTTAGCAAAGGAGAAGAGGATAACATGGCAATTATAAAAGAATTCATGAGATTTAAGGTTCATATGGAAGGTTCTGTAAATGGACACGAGTTTGAAATTGAAGGAGAAGGTGAAGGAAGGCCATACGAAGGGACACAAACTGCAAAATTAAAAGTTACTAAAGGTGGACCTCTCCCATTCGCATGGGATATTTTATCTCCTCAATTTATGTATGGATCTAAGGCATACGTCAAACATCCAGCAGATATCCCGGATTACTTGAAACTTAGTTTTCCAGAAGGTTTTAAATGGGAAAGGGTTATGAATTTCGAGGACGGAGGTGTGGTTACTGTGACGCAAGATTCAAGTCTACAGGATGGTGAATTTATTTATAAGGTAAAATTAAGAGGCACTAATTTTCCTTCCGATGGGCCCGTAATGCAAAAAAAGA |  |
|  | **HAP2 promoter** | | | 339 | GAAAATCAAACTATTAATTTAGAGATTGATTAAATAATTTTTAATTGACATAAAGTTTGTTTAAAGAATATTATTAAAAAATAAGGAATTGGATAAAGTGATATGATGAGTATTTAACAGTATCAGCTAAAATAATTAAAGTTATGGAGGAAAATCTATAAATATTTTTATTTGTTAATATGACCACACAAATAATAAGAGAATAATTAAAAAGTAAAATAGTGTGGGAGTTAAAAAAAGAATAAGTCGTTATGAAAGATATATAAATTTATATGTAATGGTTTAAAATTGAGAGAAAATTTAAAAATTTAAGATGAAAGAAGAATAAAATAATATATA |  |
|  | ***GB_8.1*** | | | 387 | GGCTGGACTAATGTTAATATTGTAATGAACTACATTAGAAATTTGGACTCTGAGACTGGGGCCATGGTTAGGTTGCTAGAGGATGGGGACTAATAATAAGTTCGTGGCGTGTAGGATAGTATCGATTAAGTTTTTCTTTTTATCTTAATTAATACTAATTCTTTTGAATTTCCACATATCTAATCATAATACTAGTCTCTAACATATAAGACTCATTTCTCTGGAGAAAGGTACATATATATTGGAGCAATTCATTATGGGATTCACACTCCCTCTACCCAATATTATCAATAATACAGATAATCAACAATTAGTAGTAGCATTTCTAAATGGTCTACTAGCAGGAATATGTCATTAAATTTTCTAACAATCTATTAAATTATTGTG |  |
|  | ***GB_8.2*** | | | 400 | AATAATACAGATAATCAACAATTAGTAGTAGCATTTCTAAATGGTCTACTAGCAGGAATATGTCATTAAATTTTCTAACAATCTATTAAATTATTGTGATTCTCTAAGATATTTGTGTTCCACTTATTTTATTAAAAACTAATATTTTTACTTGGAAGTATTTTCTTTTGCTATTTCTTAACTCAGATATACCATTCCCTACGACTTGTGAAATATTTTACTAATACTTAACTAAAACTATACAAGATTTTTTCCCACTCAAGTTCTAATCAATGCTTACTTTAAACACGTTTTTTTACACCTATATATACTTTAATATTTGTTTTCAATATTTTTATTTATTTGCATGCAAATTATATATCTAAAAATGGCGCCACAATGGCTGGTATATAATATCCAG |  |
|  | ***GB_8.3*** | | | 331 | ATTTATTTGCATGCAAATTATATATCTAAAAATGGCGCCACAATGGCTGGTATATAATATCCAGCCTGCGAGCATGCTTCAGATTTAAAGGAAAAAGGGAGAAACAGCACAAAATTTGTACTTGTTAGAATCGCAAATAAATTTTGTTAAAAAAATTTGATAAAGTAGAAATTGGTGAAAAATCTCATTTTATTTGTTTCAAAAGAAAAAAATAACTTCGTATAGCATACATTATACGAAGTTATATGGTTAGCAAAGGAGAAGAGGATAACATGGCAATTATAAAAGAATTCATGAGATTTAAGGTTCATATGGAAGGTTCTGTAAATGG |  |
